# Curcumin-Loaded Polysaccharide Nanoparticles Enhance Aqueous Dispersibility and In Vitro Cytotoxicity in Breast Cancer Cell Lines

**DOI:** 10.3390/nano15221747

**Published:** 2025-11-20

**Authors:** Yu-Chen Tsai, Hiroki Miyajima, Ming-Yang Chou, Satoshi Fujita

**Affiliations:** 1 Department of Frontier Fiber Technology and Sciences, University of Fukui, Fukui 910-8507, Japanh.miyajm@u-fukui.ac.jp (H.M.); 2 ROHER Technology Co., Taichung 41141, Taiwan, China

**Keywords:** curcumin, nanoparticle, chitosan, hyaluronic acid, alginate, cytotoxicity, anticancer, breast cancer, nanocarrier

## Abstract

Curcumin (CUR) is a natural compound with anticancer potential; however, its poor water solubility, instability, and rapid degradation limit its therapeutic use. To address these issues, we developed CUR-loaded nanoparticles (CUR-NPs) based on chitosan, hyaluronic acid, and alginate, the TEM-measured diameter of 29.3 ± 9.0 nm. Dynamic light scattering (DLS) analysis further confirmed good aqueous dispersibility, revealing hydrodynamic diameters of 39.8 ± 7.1 nm for UL-NPs and 46.1 ± 18.1 nm for CUR-NPs. Cytotoxicity assays revealed significant anticancer activity in both MCF-7 and MDA-MB-231 cells, with IC_50_ values of 17.5 ± 1.9 μg/mL and 39.9 ± 5.4 μg/mL after 72 h, respectively, indicating cell line-dependent sensitivity with MCF-7 cells being more susceptible to CUR-NP treatment. Time-dependent uptake was confirmed using fluorescence imaging and flow cytometry, which demonstrated faster and higher NP uptake by MCF-7 cells than by MDA-MB-231 cells. Collectively, these data support a cell line-dependent cell death response: MCF-7 cells displayed earlier and more pronounced changes consistent with apoptosis, whereas MDA-MB-231 cells showed slower uptake with delayed apoptosis and partial necrosis. Subcellular localization dynamics, particularly perinuclear aggregation, have emerged as determinants of NP-induced cytotoxicity, highlighting the potential for tailoring NP design to specific cellular contexts to improve therapeutic efficacy.

## 1. Introduction

The global cancer burden continues to rise, and the World Health Organization’s Cancer Agency predicts that by 2050, the number of diagnoses and deaths from breast cancer will increase significantly. Breast cancer will remain the most commonly diagnosed cancer among women and one of the leading causes of cancer-related deaths [1,2,3]. Metastasis accounts for approximately 90% of cancer-related deaths and is the primary cause of cancer-related mortality [4]. Although current clinical treatments commonly include surgery, radiation therapy, and chemotherapy [5,6], their efficacy is limited by side effects and drug resistance. Therefore, novel therapeutics, including natural medicines and innovative delivery systems, should be urgently explored as alternatives or adjunctive approaches.

Breast cancer can be classified into three major subtypes based on the expression of tumor cell proteins: hormone receptor-positive (estrogen receptor and/or progesterone receptor, ~70%), HER2-positive (~15–20%), and triple-negative breast cancer (TNBC, ~15%). TNBC is highly aggressive, frequently occurs in younger women, and lacks hormone receptor and HER2 expression. Although initially responsive to chemotherapy, its overall prognosis remains poor [7,8].

In recent years, the integration of natural medicines with polymeric materials has emerged as a promising strategy for treating cancer. Natural medicines exert diverse anticancer effects, and natural polymeric biomaterials offer excellent biodegradability and favorable drug carrier properties, improving drug stability, targeting efficiency, and therapeutic efficacy [9,10]. Chitosan (CS) is one of the most common naturally occurring non-toxic biopolymers and is obtained via partial N-deacetylation of chitin. Owing to its biocompatibility, biodegradability, low toxicity, and mucoadhesive capacity, CS has attracted increasing attention in the food and biomedical industries since it was first identified in the 19th century [11] with applications as an antibacterial [12], antioxidant [13], antihypertensive [14], antitumor [15], and wound dressing agent [16]. CS nanoparticles (NPs) demonstrate excellent drug delivery and tumor-targeting capabilities and have been extensively investigated as anticancer drugs, gene therapeutics, and antiviral agents, establishing CS as one of the most promising nanocarriers among natural polymeric materials [17].

Despite these advantages, CS often exhibits insufficient colloidal stability, which restricts its use as a standalone drug delivery system [18]. Combining CS with negatively charged alginate (ALG) yields a complementary composite that suppresses aggregation and enables pH-sensitive release [19,20], whereas the incorporation of hyaluronic acid (HA) can impart active targeting via CD44 binding on cancer cell surfaces, thereby enhancing targeting specificity [21,22]. Notably, the diameters of most reported polysaccharide-based NPs exceed 100 nm, and they become unstable within several weeks, limiting deep tumor penetration and long-term usability. In contrast, ultrasmall NPs (<50 nm) can penetrate tumor tissues more efficiently and accumulate near the perinuclear region, thereby facilitating enhanced intracellular delivery and antitumor efficacy. Therefore, achieving a stable ultrasmall particle size is a crucial design objective.

Curcumin (CUR), a natural polyphenolic compound derived from the turmeric rhizome (*Curcuma longa*), has been used in Asian medicine for centuries [23]. Modern pharmacology has confirmed its anti-inflammatory [24], antioxidant [25], antibacterial [26], and antiviral [27] activities. Its ability to inhibit tumor cell proliferation and induce apoptosis has poised it as a promising anticancer candidate [28]. CUR has a favorable safety profile and is generally recognized as safe by the Food and Drug Administration, USA [29]. However, its clinical translation remains challenging due to poor aqueous solubility, chemical instability, and rapid metabolism in the gastrointestinal tract and liver, which together result in extremely low systemic bioavailability [30,31,32]. For instance, even at oral doses of up to 10 g, the peak serum concentration does not exceed 50.5 ng/mL [31,32]. These limitations underscore the need to develop advanced drug delivery strategies to improve CUR solubility, stability, and bioavailability.

To address these challenges, a high drug encapsulation efficiency (EE%) is required to maximize CUR loading and minimize loss during formulation. Electrostatic interactions between positively charged CS and negatively charged ALG or HA can improve CUR entrapment and sustained release, while maintaining NP stability under physiological conditions. Nanocarrier technology thus ensures improved solubility, controlled release, and targeted delivery; nevertheless, long-term stability remains a bottleneck as aggregation and drug leakage occur often during storage. Developing a formulation that retains physicochemical integrity for several months under ambient conditions is a significant step toward clinical translation. Extending the circulation time has also become a key strategy to enhance the use of natural medicines [33].

Therefore, in this study, we aimed to utilize an NP drug delivery system to enhance the water solubility and therapeutic potential of CUR. Unlike most studies that have used single-cell models, we simultaneously used MCF-7 and MDA-MB-231 cells to compare the uptake efficiency, cytotoxicity, and therapeutic effects of CUR. We believe that high CUR encapsulation efficiency and extended stability will provide a robust and scalable nanocarrier platform for enhanced breast cancer therapy.

## 2. Materials and Methods

### 2.1. Materials

The MCF-10A and MDA-MB-231 human breast cancer cells and MCF-7 human mammary epithelial cells were obtained from the American Type Culture Collection (ATCC). Dulbecco’s modified Eagle’s medium (DMEM) was purchased from Sigma-Aldrich (Tokyo, Japan). Ethanol, paraformaldehyde, Hoechst 33342, hydrocortisone (cat. no. 086-02484), recombinant human epidermal growth factor (cat. no. 058-09521), and recombinant human insulin (cat. no. 099-06473) were purchased from FujiFilm Wako Pure Chemical (Tokyo, Japan). Phosphate-buffered saline (PBS) and SF cell count reagent were obtained from Nissui Pharmaceutical (Tokyo, Japan) and Nacalai Tesque (Kyoto, Japan), respectively.

CUR, CUR-loaded NPs (CUR-NPs), and unloaded NPs (UL-NPs, drug-free) were purchased from Roher Technology Co. (Taichung, Taiwan). The NPs used in this study were prepared via an ionic-gelation method, following methodologies similar to those previously reported with minor modifications [34,35,36]. In this process, CS, ALG, and HA were used as the primary biopolymeric constituents. In total, 27 g of these biopolymers was used as the foundational matrix for NP fabrication, yielding a carrier system, hereafter designated as CS/ALG/HA NPs. This formulation was designed to utilize the biocompatibility of CS, the polyanionic characteristics of ALG, and the tumor-targeting properties of HA, thereby enhancing the physicochemical stability and biological performance of NPs. The resulting NP formulations were further distinguished based on the quantity of CUR incorporated during the synthesis process, with the specific sample codes assigned as listed in Table 1.

### 2.2. Characterization of Prepared NPs

The surface morphology and particle size of the NPs were characterized using scanning electron microscopy (SEM) and transmission electron microscopy (TEM). For SEM analysis, the NPs were diluted with deionized (DI) water and the suspension was air-dried at the sample stage. The dried samples were then coated with osmium using a sputter-coating system (MSP-1S; Vacuum Device, Ibaraki, Japan) and imaged using a scanning electron microscope (JSM-7800F; JEOL, Tokyo, Japan) operated at 5 kV. For the TEM observations, the NP suspensions were dropped onto copper grids (ELS-C10 STEM Cu100P; Okenshoji Co., Ltd., Tokyo, Japan), air-dried, and examined using TEM (H-7650; Hitachi Ltd., Tokyo, Japan) at an acceleration voltage of 80 kV. Particle size distributions were determined from the TEM images using ImageJ (version 1.54p). At least 120 individual particles were measured per sample, and the results are presented as histograms showing the mean diameter ± standard deviation (SD). To evaluate long-term stability, SEM and TEM images were obtained at storage intervals of 0, 21, 35, 110, and 195 days under identical conditions at room temperature, for each stability time point, at least 100 nanoparticles were measured to allow enabling the comparison of NP morphology and particle size over time.

In addition, the NPs were appropriately diluted with DI water prior to dynamic light scattering (DLS) measurements to determine their hydrodynamic size, polydispersity index (PDI), and zeta potential. DLS analyses were conducted at 25 °C using (SZ-100, HORIBA, Kyoto, Japan), with each measurement performed in triplicate.

Fourier transform infrared (FTIR) spectroscopy was performed to verify the successful encapsulation of CUR. The FTIR spectra of free CUR, ALG, HA, and both CUR-NPs and UL-NPs were recorded using an ATR-FTIR spectrometer (Thermo Fisher Scientific, Madison, WI, USA) over a range of 400–4000 cm.

### 2.3. EE% and Drug Loading (DL%) of the Prepared NPs

CUR-NPs were prepared and quantified as described previously [34] with minor modifications. Briefly, the NPs were diluted in DI water and analyzed using a UV–vis spectrophotometer (UV-2900; Hitachi, Tokyo, Japan) in the range 300–600 nm. Enzymatic degradation was performed using a commercial cellulase-containing enzyme blend (cat. No. 83502; Deerland Probiotics & Enzymes, Kennesaw, GA, USA). After incubation with the enzyme, CUR was released, dissolved in dimethyl sulfoxide (DMSO), and quantified at 433 nm using a standard calibration curve (0–1 mg/mL). EE% and DL% were calculated as the proportion of encapsulated CUR relative to the initial CUR and total NP mass, respectively (Equations (1) and (2)).

(1) $$EE\%=\frac{Amount\;of\;CUR\;in\;the\;CUR\;NPs}{Initial\;mass\;of\;CUR}\times 100$$

(2) $$DL\%=\frac{Amount\;of\;CUR\;in\;the\;CUR\;NPs}{Total\;mass\;of\;the\;CUR-NPs\;}\times 100$$

The CUR concentration in the extracted samples was determined by comparison with the calibration curve shown in Figure S1. The absorbance values obtained from the UV–Vis spectrophotometer were converted to CUR concentrations, and dilution factors were applied to calculate the total mass of encapsulated CUR. EE% and DL% were then calculated according to Equations (1) and (2).

### 2.4. Cell Culture

MCF-10A cells were cultured in DMEM supplemented with 10% fetal bovine serum (FBS; Atlas Biologicals Inc., Fort Collins, CO, USA), 20 ng/mL EGF, 10 μg/mL insulin, and 0.5 μg/mL hydrocortisone (Fujifilm Wako Pure Chemical Corporation, Osaka, Japan). MDA-MB-231 and MCF-7 cells were cultured in DMEM supplemented with 10% FBS, 1% L-glutamine (Life Technologies Corporation, Grand Island, NY, USA), and 1% penicillin/streptomycin (Nacalai Tesque, Kyoto, Japan). All cultures were maintained at 37 °C in a humidified atmosphere containing 5% CO_2_. All experiments were performed with cells counted and seeded at equal initial densities.

### 2.5. Cell Viability Assay

The cytotoxicity of the CUR1.4-NPs was assessed using the WST-8 assay (Cell Count Reagent SF kit; Nacalai Tesque, Kyoto, Japan). Cells were seeded at a density of 1 × 10^4^ cells/well in 96-well plates and cultured in a maintenance medium containing 10% FBS. After overnight incubation at 37 °C in an atmosphere of 5% CO_2_ for cell attachment, the cells were treated with free CUR (208 μg of CUR dispersed in 1 mL of DI water) or CUR1.4-NPs (final concentrations, 13, 26, 52, 104, and 208 μg/mL) for 24, 48, or 72 h. After treatment, 10 μL of reagent solution was added to each well and incubated for 2 h. Absorbance was then measured at 450 nm using a microplate reader (Multiskan GO; Thermo Fisher Scientific, Waltham, MA, USA).

To determine the 50% inhibitory concentration (IC_50_), a calibration curve was generated using free CUR dissolved in DMSO. The NPs were disrupted to quantify the encapsulated CUR and the obtained values were plotted on the calibration curve. Therefore, the concentrations used in the cytotoxicity assay represent the final CUR concentration in the NP suspension and the IC_50_ (μg/mL) refers to the mass of CUR.

### 2.6. Fluorescein CUR-NP Conjugates

Fluorescein-conjugated CUR-NPs (Fl-CUR-NPs) were prepared as described previously [37] using CUR1.4-NPs instead of AC8-NPs. Briefly, 1 mL of CUR1.4-NP suspension in PBS (pH 7.4) was reacted with 10 µL of NHS fluorescein solution (0.1 M in DMSO) for 2 h at room temperature in the dark. After repeated centrifugation and washing with PBS, the resulting Fl-CUR-NPs were examined using fluorescence microscopy (Olympus IX-81; Olympus Optical Co., Ltd., Tokyo, Japan).

### 2.7. Fluorescent Microscopy

Cells (1 × 10^5^) were seeded in 35 mm glass-bottom dishes containing 2 mL of DMEM (with 10% FBS) and cultured overnight at 37 °C. The following day, the cells were exposed to Fl-CUR-NPs (50 μL, original concentration 1.3 mg/mL, equivalent to a final CUR concentration of ~0.032 mg/mL) for 0.5, 1, 3, 6, or 24 h. After incubation, the residual NPs were removed using PBS, and the cells were fixed with 4% paraformaldehyde. The fixed samples were washed with PBS and stained with Hoechst 33342 (1:2000 dilution in PBS; Dojindo, Kumamoto, Japan) for 15 min at room temperature in the dark. The intracellular fluorescence distribution and accumulation of NPs were visualized using a fluorescence microscope. Untreated cells were used as negative controls.

### 2.8. Flow Cytometric Quantification

For quantitative analysis of NP uptake, 2 × 10^5^ cells/well were seeded in 6-well plates and allowed to adhere overnight. The cells were then treated with 50 μL of Fl-CUR-NPs (1.3 mg/mL; final CUR concentration ~ 0.032 mg/mL) and incubated for 0.5, 1, 3, 6, or 24 h. Untreated cells were included as controls for background correction. At each time point, the cells were washed twice with PBS to remove extracellular particles, detached using trypsin, and resuspended in PBS. Fluorescence intensity was detected using a FACSymphony A1 flow cytometer (Becton Biosciences, San Jose, CA, USA). Forward scatter and side scatter parameters were applied to gate viable cells and to exclude debris. At least 10,000 gated events per sample were analyzed to ensure statistical reliability.

### 2.9. Statistical Analysis

All data are presented as mean ± standard deviation (SD) from three independent experiments. Statistical comparisons among the groups were performed using Tukey’s post hoc test. Significance levels were set at * *p* < 0.05 and ** *p* < 0.001.

## 3. Results

### 3.1. Characterization of CUR-NPs

#### 3.1.1. Surface Morphology and Particle Size of Synthesized CUR-NPs

**Figure 1:**
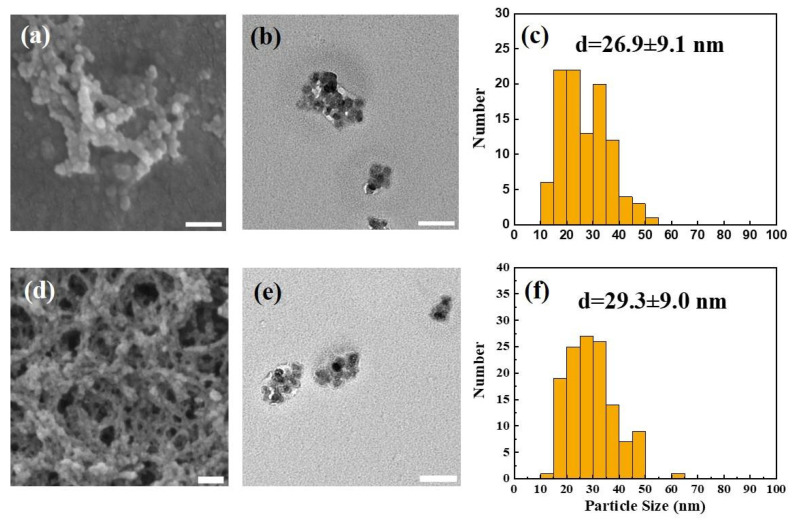
Morphological characterization of NPs immediately after preparation using SEM, TEM, and particle size distribution analysis. (**a**–**c**) UL-NPs; (**d**–**f**) CUR-NPs. All scale bars = 100 nm.

As shown in Figure 1, the SEM and TEM images revealed that the NPs prepared in this study exhibited regular spherical and visually homogeneous morphologies with no obvious structural abnormalities, indicating the excellent controllability and reproducibility of the fabrication process (Figure 1a,b,d,e). Particle size analysis revealed that the average diameter of the UL-NPs was 26.9 ± 9.1 nm (Figure 1c), which slightly increased to 29.3 ± 9.0 nm after encapsulating CUR (Figure 1f). This indicates that DL% changed particle size negligibly and that the overall uniformity was well maintained. This aligns with the expected trend of drug molecule loading within the NPs and indirectly confirms the success of the encapsulation process.

In addition, DLS characterization further confirmed the nanoscale hydrodynamic sizes of both UL-NPs (39.8 ± 7.1 nm; PDI = 0.43) and CUR-NPs (46.1 ± 8.1 nm; PDI = 0.49), indicating moderate dispersity in aqueous media. Zeta potential analysis revealed positively charged surfaces for UL-NPs (+36.2 mV) and CUR-NPs (+35.3 mV), shown in Table 2**,** which are favorable for colloidal stability and cellular interactions.

**Table 2:** Physicochemical characteristics of UL-NPs and CUR-NPs.

Sample	SEM/TEM Diameter (nm)	DLS Hydrodynamic Size (nm)	PDI	Zeta Potential (mV)
UL-NPs	26.9 ± 9.1	39.8 ± 7.1	0.43	+36.2
CUR-NPs	29.3 ± 9.0	46.1 ± 8.1	0.49	+35.3

#### 3.1.2. Long-Term Morphological Stability of CUR-NPs

**Figure 2:**
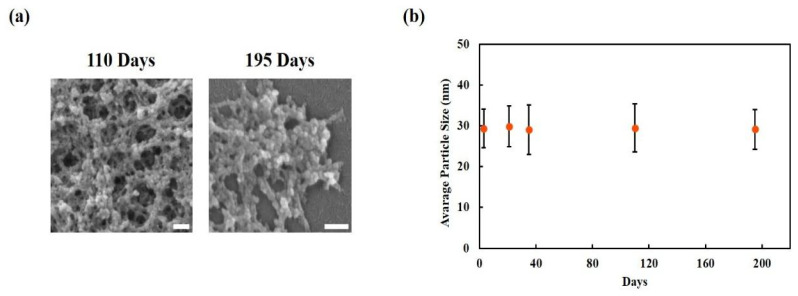
Morphological stability of CUR-NPs during storage. (**a**) Representative SEM images of NPs after 110 and 195 days of storage. (**b**) Particle size measurements at 0, 21, 35, 110, and 195 days. No significant difference was observed compared with day 0. Data are presented as mean ± SD (*n* = 3). Scale bars = 100 nm. Red dots represent the mean particle size measured at each time point (0, 21, 35, 110, and 195 days), and black error bars indicate the standard deviation.

To evaluate the long-term morphological stability of the NPs, CUR-NPs were stored at room temperature and subjected to SEM and TEM analyses after different storage durations (0, 21, 35, 110, and 195 days). SEM images confirmed that the CUR-NPs maintained regular spherical structures with no apparent disintegration at 110 and 195 days (Figure 2a). Particle size measurements did not reveal any significant differences in the average particle size compared to that observed on day 0 throughout the 195-day storage period, indicating a stable particle size distribution over the entire observation period (Figure 2b). Additionally, particle size distribution profiles for each storage time point (Figure S2) further confirmed that no notable broadening in size distribution occurred during long-term storage.

Furthermore, to confirm whether storage affected the biological performance of the nanoparticles, cellular uptake was re-evaluated after three months of storage. As shown in Supplementary Figure S3, both MCF-7 and MDA-MB-231 cells exhibited comparable fluorescence uptake patterns between freshly prepared and stored Fl-CUR-NPs, indicating preserved internalization efficiency and functional stability.

In addition to morphological evaluation, the chemical stability and drug retention of CUR within the nanoparticles during storage were further investigated using UV–Vis spectroscopy. As shown in Supplementary Figure S4, the absorption profiles of CUR-NPs remained highly consistent over time, with the characteristic peaks of CUR (~425–450 nm) clearly preserved after 3 and 60 days of storage. No noticeable shift in absorbance intensity was observed compared with freshly prepared CUR-NPs, suggesting that CUR remained chemically stable without degradation or leaching from the carrier system. These results indicate that the CUR-NPs retained their structural integrity and drug content during long-term storage.

#### 3.1.3. Structural Characterization of CUR-NPs Using FTIR Analysis

**Figure 3:**
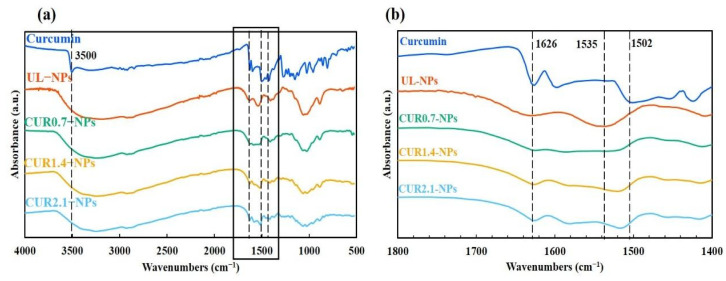
(**a**) FTIR spectra of free CUR, UL-NPs, and CUR-NPs. (**b**) The enlarged region of the characteristic peaks (1400–1800 cm^−1^) is shown for better visualization. Vertical dotted lines indicate the characteristic peaks of curcumin, and the black frame highlights the magnified region of these characteristic peaks.

FTIR analysis was performed to investigate CUR encapsulation. The characteristic peaks of the FTIR spectra for each raw material, shown in Figure S5, are consistent with those reported in the literature [38,39,40] and act as reference benchmarks for subsequent comparisons of NP characteristics. In the CUR spectrum (Figure 3a), a prominent peak at 3500 cm^−1^ corresponds to the hydroxyl functional group (–OH). The peak at 1626 cm^−1^ primarily originates from vibrations of carbon–carbon double bonds (C=C) and carbonyl groups (C=O), while the intense 1597 cm^−1^ peak is attributed to symmetric stretching vibrations of aromatic ring carbon–carbon bonds (C=C). The peak at 1425 cm^−1^ corresponds to C=C stretching vibrations, while the peak at 1502 cm^−1^ is associated with CUR’s characteristic carbonyl (C=O) vibrations and aromatic ketone/enol groups [40,41]. In the UL-NP spectrum, absorption peaks at 1377 and 1535 cm^−1^ correspond to the N–H bending vibration of amide II and the strain vibration of the CS–NH_2_ functional group, respectively. Additionally, the prominent peak at 1029 cm^−1^ belongs to the C–O–C stretching vibration of the HA polysaccharide backbone, representing one of its characteristic absorption peaks. ALG exhibits characteristic peaks at 1630 and 1405 cm^−1^, corresponding to the asymmetric and symmetric stretching vibrations of the COO^−^ group, respectively. These characteristic absorption peaks are consistent with previously reported results for CS, HA, and ALG, further supporting the composition and structural integrity of the NPs prepared in this study [37].

To observe subtle changes in functional group peak positions more clearly, a specific region (1400–1800 cm^−1^) was magnified, as shown in Figure 3b. In the CUR-NP spectrum, a broad peak near 1630 cm^−1^ gradually intensified with increasing CUR concentration (CUR0.7–NPs to CUR2.1–NPs). Additionally, a distinct peak shift occurred at 1535 cm^−1^, progressively red-shifting due to molecular interaction with increasing encapsulation concentration.

#### 3.1.4. EE% and DL% Capacity of CUR-NPs

**Figure 4:**
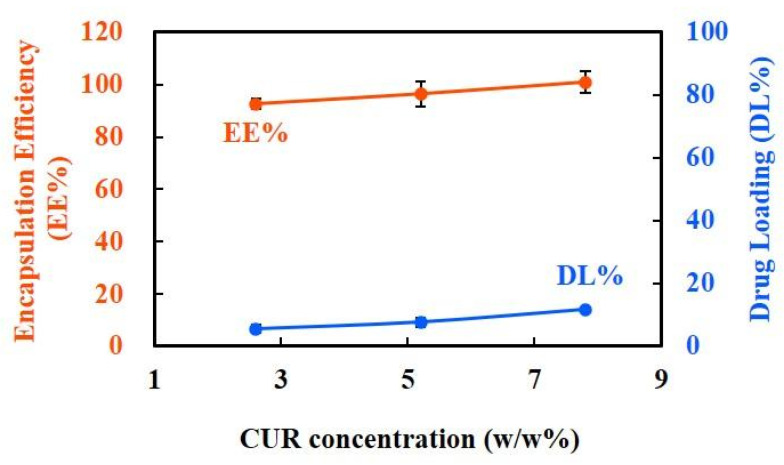
EE% and DL% of CUR-NPs at different CUR concentrations. The results indicate that the prepared NPs exhibit consistently high encapsulation efficiency (approximately 90–100%) across all tested conditions, while drug loading gradually increases (from ~5% to ~12%) with higher CUR concentrations, demonstrating excellent encapsulation ability and concentration-dependent drug-loading capacity. Data are presented as mean ± SD (*n* = 3). Red dots represent the mean encapsulation efficiency (EE%) at each curcumin concentration. Blue dots represent the mean drug loading (DL%) at each curcumin concentration. Black lines indicate the corresponding error bars.

As shown in Figure 4, the prepared CUR-NPs exhibited extremely high EE% (approximately 90–100%) across different CUR concentrations, indicating that the carrier system effectively captured and immobilized the drug molecules. In contrast, DL% gradually increased with CUR concentration (approximately 5% to 12%), reflecting the excellent drug-loading capacity and concentration dependency of NPs. Overall, this process not only ensures highly efficient drug encapsulation but also enables flexible dose design by controlling the concentration. Quantification accuracy was validated using a standard calibration curve (R^2^ > 0.99) provided in Figure S1, ensuring reliable determination of CUR content in all formulations.

### 3.2. In Vitro Biological Evaluation of CUR-NPs

#### 3.2.1. Cytotoxicity

**Figure 5:**
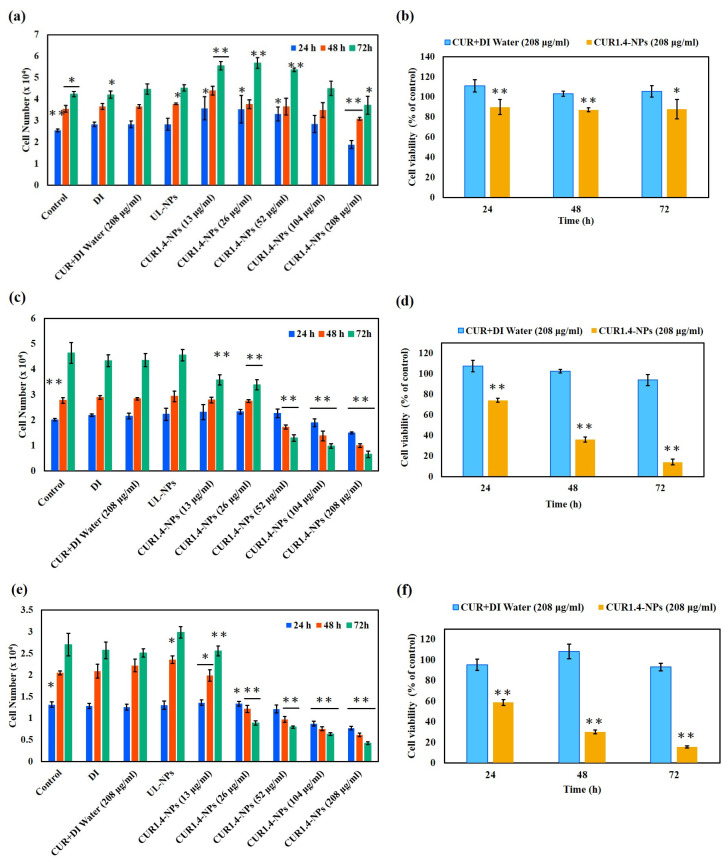
Cytotoxicity of free CUR and CUR1.4-NPs in breast cell lines. Cell viability of (**a**) MCF-10A, (**c**) MDA-MB-231, and (**e**) MCF-7 after 24–72 h of treatment with CUR + DI water or CUR1.4-NPs. Comparisons at 208 µg/mL are shown in (**b**,**d**,**f**). Data are presented as mean ± SD (*n* = 6). Statistical analysis was performed using Tukey’s post hoc test. * *p* < 0.05 and ** *p* < 0.001 vs. CUR + DI water group at the same time point. Black lines indicate the corresponding error bars.

Figure 5a shows the cytotoxicity of the formulation in MCF-10A normal breast epithelial cells. These results clearly indicate that different concentrations of NPs exhibit negligible cytotoxicity. At all test concentrations and incubation periods, the cells maintained good growth, and cell viability was slightly higher than that of the control group even at low concentrations, indicating that this nanosystem has good biocompatibility and is safe for normal cells. As shown in Figure 5b, cell viability remained above 80% even at the highest NP concentration, further confirming the excellent cytocompatibility of the formulation.

Figure 5c shows the cytotoxic effects of the same treatment on MDA-MB-231 cells. The IC_50_ value calculated after 72 h of treatment was 39.9 ± 5.4 µg/mL, confirming the time- dependent and concentration-dependent inhibitory effect. Notably, at the equivalent concentration of 208 µg/mL, even after 72 h, the CUR + DI water group (free CUR dispersed in distilled water) still maintained 93.9% cell viability (Figure 5d), indicating its limited cytotoxicity. In sharp contrast, the cell viability of the CUR1.4-NP group sharply decreased to approximately 14% after 72 h, showing a significant time-dependent cytotoxic response. In other words, the cytotoxic effect of CUR1.4-NPs was approximately 6.7 times that of CUR + DI water, highlighting the significant contribution of encapsulation in enhancing drug efficacy.

Similarly, Figure 5e shows the cytotoxicity of MCF-7 breast cancer cells; its IC_50_ value of 17.5 ± 1.9 µg/mL at 72 h was significantly lower than the value observed in MDA-MB-231 cells. Consistent with the results obtained using MDA-MB-231, the effects of the same concentration of CUR + DI water and CUR1.4-NPs (208 µg/mL) over different incubation periods showed a similar trend (Figure 5f). The cytotoxicity of the CUR1.4-NPs was significantly higher than that of free CUR, further verifying the superior therapeutic effects of the NP formulation.

#### 3.2.2. Cellular Uptake Behavior and Morphological Alterations of CUR-NPs

**Figure 6:**
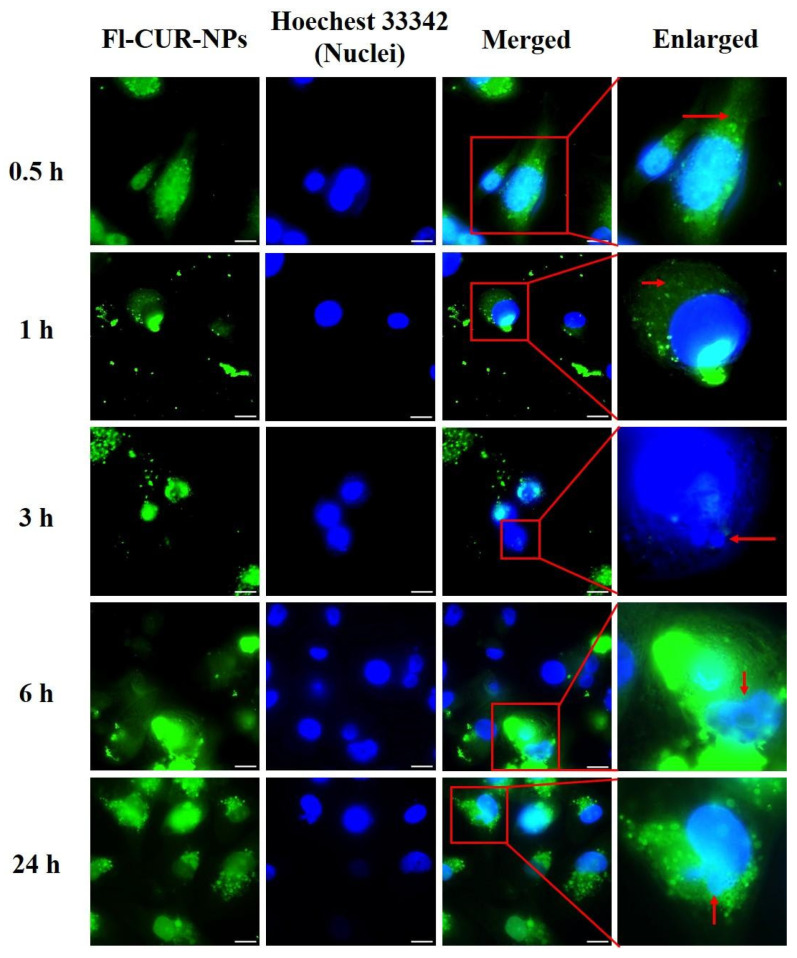
Fluorescence microscopy images of MDA-MB-231 incubated with Fl-CUR-NPs at 37 °C for 0.5, 1, 3, 6, and 24 h. Green fluorescence represents captured Fl-CUR-NPs and blue fluorescence indicates nuclei stained with Hoechst 33342. Red arrows indicate regions showing notable cellular morphological changes. Scale bars = 20 μm.

**Figure 7:**
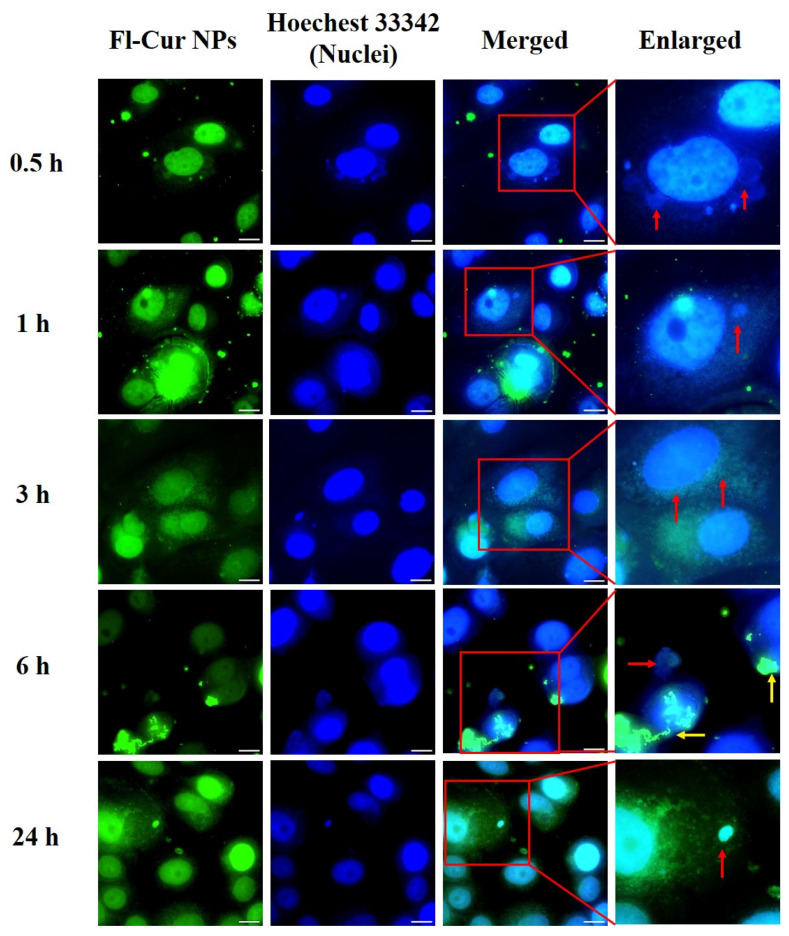
Fluorescence microscopy images of MCF-7 incubated with Fl-CUR-NPs at 37 °C for 0.5, 1, 3, 6, and 24 h. Green fluorescence indicates the captured Fl-CUR-NPs and blue fluorescence indicates nuclei stained with Hoechst 33342. Scale bars = 20 μm. Red arrows indicate pronounced organelle extrusion, while yellow arrows highlight partial Fl-CUR-NP release.

**Figure 8:**
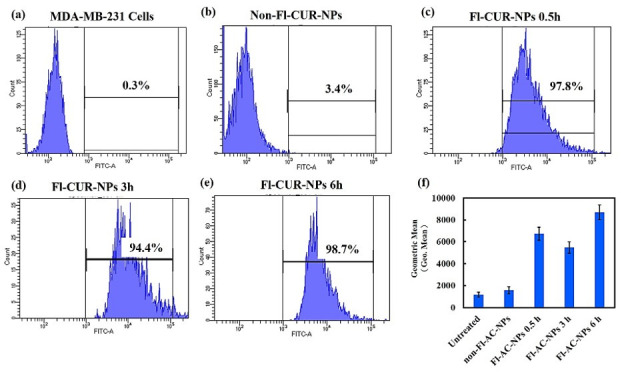
Cellular uptake of Fl-CUR-NPs by MDA-MB-231 analyzed using flow cytometry. (**a**) Untreated control cells, (**b**) cells treated with non-fluorescent CUR-NPs, and (**c**–**e**) cells treated with Fl-CUR-NPs for 0.5, 3, and 6 h, respectively. (**f**) Quantitative analysis of mean fluorescence intensity at each time point, reflecting the relative amount of NPs incorporated by each cell. The black line in panels (**a**–**e**) represents the percentage of fluorescence intensity, whereas the black line in panel (**f**) indicates the error bars.

**Figure 9:**
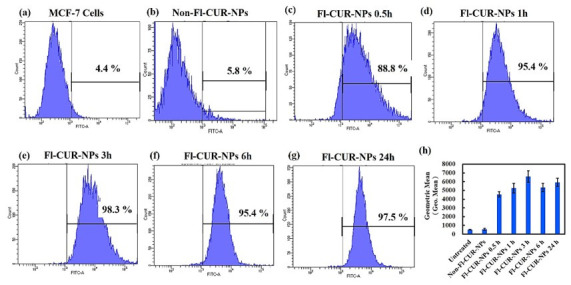
Cellular uptake of Fl-CUR-NPs by MCF-7 analyzed using flow cytometry. (**a**) Untreated control cells, (**b**) cells treated with non-fluorescent CUR-NPs, and (**c**–**g**) cells treated with Fl-CUR-NPs for 0.5, 1, 3, 6, and 24 h, respectively. (**h**) Quantitative analysis of mean fluorescence intensity at each time point, reflecting the relative amount of NPs incorporated by each cell. The black line in panels (**a**–**g**) represents the percentage of fluorescence intensity, whereas the black line in panel (**h**) indicates the error bars.

The cellular uptake and morphological alterations of CUR-NPs in MDA-MB-231 and MCF-7 cells were observed using fluorescence microscopy and confirmed using flow cytometric analysis at various time points (Figure 6, Figure 7, Figure 8 and Figure 9). Both cell types exhibited rapid cellular uptake of NPs, as fluorescence signals were clearly detected in the cytoplasm as early as 0.5 h post-treatment (Figure 6 for MDA-MB-231; Figure 7 for MCF-7).

##### Visualization of CUR-NP Uptake and Morphological Changes Using Fluorescence Microscopy

At 0.5 h, MDA-MB-231 cells retained their typical spindle-like morphology, and Fl-CUR-NPs were clearly distributed within the cytoplasm. After 1 h, the cells gradually became rounded with reduction in cytoplasmic volume, whereas Fl-CUR-NPs remained visible in the cytoplasm. At 3 h, partial cell rupture and extrusion of intracellular organelles were observed, and some Fl-CUR-NPs were distributed around the nuclear region or released from the cells. At 6 h, prominent cytoplasmic compression and increased membrane permeability were observed, accompanied by continuous Fl-CUR-NP release. After 24 h, most cells displayed a rounded morphology, with numerous Fl-CUR-NPs dispersed in the cytoplasm, and nuclear fragmentation was frequently observed.

At 0.5 h, Fl-CUR-NPs were observed predominantly in the cytoplasm of MCF-7 cells, with fluorescence signals frequently appearing near the nuclear region vesicle-like structures surrounding the nucleus. After 1 h of exposure, the cells began to display morphological deformation and nuclear compression along with partial extrusion of cytoplasmic components from the perinuclear area. After 3 h, morphological features consistent with cell stress, including membrane blebbing, cell shrinkage, and formation of small vesicle-like fragments were more apparent. After 6 h, pronounced organelle extrusion (red arrows) and partial Fl-CUR-NP release (yellow arrows) were observed. After 24 h, numerous vesicle-like structures and fragmented nuclei were visible near the nuclear region, indicating progressive morphological alterations after Fl-CUR-NP exposure.

##### Quantitative Analysis of Cellular Uptake Using Flow Cytometry

Flow cytometry analysis further quantified the time-dependent cellular uptake of CUR-NPs by MDA-MB-231 and MCF-7 cells (Figure 8 and Figure 9). A standardized gating strategy was applied to ensure accurate selection of single and viable cells (Figure S6), thereby minimizing debris and doublet interference. In MDA-MB-231 cells, the fluorescence intensity markedly increased at 0.5 h, indicating rapid NP cellular uptake, followed by a decrease at 3 h, suggesting partial exocytosis or NP efflux. At 6 h, the fluorescence intensity again increased, consistent with the results of microscopy, implying a secondary accumulation phase, possibly due to endosomal recycling or reuptake.

In contrast, MCF-7 cells exhibited a distinct kinetic pattern, showing a sharp increase in fluorescence at 0.5 h, reaching a plateau at 3 h, and maintaining high intracellular fluorescence for up to 24 h. A slight decline at 6 h indicated the onset of exocytosis; however, the overall high intensity was indicative of strong intracellular retention. These results were in good agreement with the fluorescence microscopy observations, confirming the efficient and sustained uptake of CUR-NPs in both cell lines.

## 4. Discussion

### 4.1. Improved Solubility and Cellular Uptake of CUR from CS/ALG/HA NPs

In the present study, we evaluated the anticancer potential of CUR-NPs. Although numerous studies have reported the anticancer activity of CUR [28,42,43], its clinical application remains limited owing to its unfavorable physicochemical properties, particularly its poor water solubility and low bioavailability [44]. Previous studies have indicated that CUR exhibits an aqueous solubility of only approximately 0.214 μg/mL [29], reflecting extremely low in vivo absorption and utilization rates. Therefore, improving the solubility and bioavailability of CUR is essential for its further pharmaceutical development.

To address these challenges, a CUR nanodelivery system was designed to enhance aqueous dispersibility and cellular uptake efficiency. These results demonstrated that this nanosystem significantly enhanced the inhibitory effects of CUR across various cancer cell types, providing a promising strategy for its application in tumor therapy. Moreover, successful encapsulation of CUR within the CS nanocarriers was confirmed. The interactions between CUR and CS primarily involved hydrogen bonding and electrostatic interactions [45], effectively overcoming the inherent limitations associated with the hydrophobic nature of CUR. The observed solubilization effect can be attributed to the abundant amino (–NH_2_) and hydroxyl (–OH) groups on CS, which form stable hydrogen-bonding networks with the phenolic hydroxyl and carbonyl groups of CUR, thereby improving its aqueous dispersion and stability [46].

### 4.2. Controlled Particle Size and Fabrication Advantages of CS/ALG/HA NPs

In this study, the mean particle size of UL-NPs was 26.9 ± 9.1 nm (Figure 1c), which increased slightly to 29.3 ± 9.0 nm after CUR encapsulation (Figure 1f), confirming successful DL%. DLS measurements further verified that both UL-NPs and CUR-NPs maintained nanoscale hydrodynamic sizes with moderate dispersity in aqueous media. Moreover, the positive zeta potentials indicated favorable colloidal stability and may contribute to enhanced cellular interaction. Notably, the particle size obtained in our formulation was substantially smaller than those reported in previous studies. For instance, a recent study optimized CS-CUR NPs with a mean size of 118 nm [39], whereas another study described CUR-loaded chitosan-based solid lipid NPs with an average diameter of 208 nm [47], indicating that the size of most CUR–CS–based systems typically fall within the 100–200 nm range. Notably, even in recent studies, CS–ALG NPs encapsulating CUR derivatives displayed particle sizes around 340 ± 14 nm [48]. In contrast, our CUR-loaded NPs exhibited an ultrasmall mean diameter of less than 30 nm. Despite the incorporation of three polysaccharides (CS, ALG, and HA) into the formulation, the resulting NPs were considerably smaller than those reported in previous studies, highlighting the efficiency and controllability of our preparation method. This remarkably reduced size provides a larger surface area, improves aqueous dispersibility, and enhances cellular uptake efficiency, thereby contributing to improved solubility, bioavailability, and anticancer efficacy. However, the solution-phase colloidal stability warrants further characterization.

### 4.3. Evaluating the Stability of CUR-NPs

Stability during storage is a crucial characteristic determining the practical application of CUR delivery systems in clinical settings as it directly affects long-term efficacy, structural integrity, and formulation safety. Therefore, we conducted a systematic assessment of the long-term stability of CUR-NPs. It is noteworthy that most previous reports on the stability of CUR or CUR-NPs were limited to short-term observations (approximately 30–90 days), and research on long-term morphology and particle size stability is limited. For instance, Hong et al. (2021) found that the particle size of hybrid NPs changed only slightly during storage at 4 °C for 30 days, with the polydispersity index and zeta potential remaining stable [49]; in contrast, in a nanoliposome system, the particle size increased from approximately 68 nm to 847.9 nm at 4 °C and 25 °C over 90 days, and the EE% decreased significantly under ambient conditions [50]. Additionally, in the nanoemulsion + CS coating system, the authors demonstrated that the retention of CUR and the changes in particle size were better than those in the uncoated system under various pH and ion strength conditions [51].

Our CUR-NPs (Figure 2a) were stored at room temperature for up to 195 days. The average particle size did not change significantly compared to that observed in day 0, and the SEM/TEM images (Figure 2b) did not show any sign of disintegration. Such long-term morphological stability is notable and indicative of storage robustness; however, further colloidal and compositional stability analyses are required to assess the translational suitability.

Furthermore, the addition of ALG in the formulation—an anionic polysaccharide containing carboxylate groups (–COO^−^)—enables electrostatic interactions with the cationic amino groups (–NH_3_^+^) on CS. This resulted in the formation of a stable polyelectrolyte complex. This structure not only aids in fixing CUR molecules within the nanocarrier but also enhances the structural stability and sustained-release properties of NPs [19,20]. Therefore, CS/ALG/HA NPs effectively encapsulated and stabilized CUR via the synergistic effects of hydrogen bonding and electrostatic and hydrophobic interactions, thereby enhancing its solubility and cellular availability in physiological environments.

### 4.4. Confirmation of CUR Encapsulation and Intermolecular Interaction

As shown in Figure 3b, a broad peak gradually appeared at around 1630 cm^−1^ as the CUR concentration in CUR-NPs increased (from CUR0.7-NPs to CUR2.1-NPs), indicating significant concentration-dependent encapsulation of CUR and high controllability. Notably, a distinct peak shift was also observed at 1535 cm^−1^, gradually red-shifting with increase in CUR encapsulation concentration, suggesting a stable and specific interaction via hydrogen bonding between CS and CUR molecules. These changes not only confirmed the successful incorporation of CUR into the NPs but also highlighted the effectiveness and reliability of the system design.

Similar peak shifts and intensity changes have been reported for other CS/ALG CUR-NPs. Arulmoorthy and Srinivasan [52] prepared CUR-loaded CS/ALG NPs in their study and observed characteristic absorption peaks at 3481–3483 cm^−1^, 1619–1637 cm^−1^, and 1425 cm^−1^, corresponding to –OH and C=O stretching vibrations. The changes in these peak positions reflected the possibility of hydrogen bonding and intermolecular interactions between CUR and the polysaccharide matrix. This phenomenon is consistent with the peak shift trend observed in this study, further supporting the hypothesis that the abundant amino and hydroxyl groups on the CS molecules can form stable hydrogen-bond networks with the phenolic hydroxyl and carbonyl groups of CUR, thereby enhancing the dispersibility and encapsulation stability of CUR in the aqueous phase.

### 4.5. High EE% and Concentration-Dependent DL% of CUR-NPs

High EE% and tunable DL% are key parameters for evaluating the performance of nanocarriers. As shown in Figure 4, the CUR-NPs prepared in this study exhibited high EE% (approximately 90–100%) at different CUR concentrations, indicating that the CS/ALG/HA NPs had high affinity and binding stability to CUR molecules. This result can be attributed to the formation of a stable hydrogen bond network between the amino (–NH_2_) and hydroxyl (–OH) groups of CS and the carbonyl and phenolic hydroxyl groups of CUR, which jointly promote the efficient embedding of CUR via electrostatic adsorption and hydrophobic interaction. In addition, DL% increased gradually with increasing CUR concentration (approximately 5–12%), indicating a concentration-dependent loading characteristic of the system, reflecting its good loading potential and formulation tunability.

Compared to these earlier reports, our system demonstrated superior encapsulation performance. Kumari et al. reported an EE ranging from 75% to 81% for poly(glycerol malate diacrylate)-conjugated CUR NPs, whereas Asadi et al. observed a DL% efficiency of 62% for CS–ALG nanocarriers [53,54]. In addition, Das et al. showed that the incorporation of PF127 into CS/ALG NPs enhanced the EE% by approximately 5–10-fold compared with that of CS/ALG NPs alone [55]. Collectively, these findings highlight that our nanoformulation achieved a markedly higher EE% than the previously reported CUR-based nanosystems, further supporting the effectiveness of our preparation strategy in maximizing drug entrapment and delivery.

### 4.6. In Vitro Cytotoxicity of CUR-NPs

Figure 5 demonstrates that CUR-loaded CS NPs (CUR1.4-NPs) markedly enhanced the cytotoxicity of CUR in breast cancer cells, while maintaining excellent biocompatibility in normal MCF-10A cells. This selective cytotoxicity suggests a preferential killing effect on tumor cells, highlighting the targeting potential of the nanosystem. Previous studies have reported that cancer cells tend to internalize CUR more efficiently than normal cells and that the resulting increase in intracellular accumulation correlates strongly with enhanced cytotoxicity. This may be attributed to the differences in the cellular membrane structure, protein composition, and metabolic activity between tumor and normal cells, which facilitate NP uptake [56,57].

Under high-concentration conditions (208 µg/mL), the CUR + DI water group retained high cell viability, indicating that free CUR, due to its inherent hydrophobicity and poor solubility, has limited cellular uptake, and consequently, reduced anticancer efficacy. In contrast, CUR1.4-NPs exhibited markedly stronger cytotoxicity due to improved aqueous dispersibility and promotion of cellular endocytosis. Notably, at equivalent concentrations, CUR1.4-NPs were significantly more effective than free CUR, underscoring the superiority of the nanoformulations in enhancing drug potency.

This enhanced activity can be attributed to several factors. The nanoscale size (~30 nm) of CUR1.4-NPs facilitated efficient endocytosis and deeper cellular penetration, resulting in increased intracellular accumulation and prolonged retention of CUR. Moreover, encapsulation within CS nanocarriers improved the stability and bioavailability of CUR, whereas the sustained-release behavior of NPs extended intracellular drug exposure, leading to a more pronounced time-dependent inhibitory effect [58]. Collectively, these findings demonstrate that CS-based nanoformulations significantly enhance cellular delivery in vitro and mitigate the physicochemical limitations of CUR; however, it is important to note that ultrasmall nanoparticles may be subject to rapid renal clearance or opsonization-mediated removal from systemic circulation, which could limit their in vivo accumulation at tumor sites. These pharmacokinetic considerations warrant further biological validation to optimize nanoparticle design and therapeutic efficacy.

Additionally, MCF-7 cells showed higher sensitivity to CUR1.4-NPs, with an IC_50_ value of 17.5 ± 1.9 µg/mL significantly lower than that in MDA-MB-231 cells (39.9 ± 5.4 µg/mL at 72 h). This difference may be related to differences in cell molecular subtypes and hormone receptor expression. MCF-7 is an estrogen receptor (ER) positive breast cancer cell, while MDA-MB-231 is a TNBC cell (ER^−^/PR^−^/HER2^−^). Previous studies have indicated that ER-positive cells are more sensitive to CUR-induced apoptosis and oxidative stress. Hu et al. [59] reported that CUR exerts a more significant inhibitory effect on ER-positive cell lines (MCF-7, T47D, and MDA-MB-415), whereas its activity is lower in ER/PR/HER2-negative cell lines (MDA-MB-231, MDA-MB-468, and BT-20). These results are consistent with those of the present study, further supporting that MCF-7 cells have a higher sensitivity to CUR1.4-NPs due to their molecular subtype characteristics and sensitivity to oxidative stress signals. In summary, the CS-coated CUR NP system not only effectively overcomes the limitations of poor solubility and low uptake efficiency of CUR but also exhibits excellent anticancer activity in breast cancer cells while maintaining safety in normal cells. These results emphasize the potential application of this nanosystem as an efficient and biocompatible CUR delivery platform for tumor treatment.

### 4.7. Confocal and Flow Cytometric Evaluation of NP Uptake Across Cell Types

The cellular responses of MDA-MB-231 and MCF-7 cells to Fl-CUR-NPs revealed distinct time-dependent uptake patterns and apoptotic progression, reflecting inherent differences in cell phenotypes and uptake mechanisms. At an early stage (0.5 h), MDA-MB-231 cells (Figure 6) retained their typical spindle-like morphology, with Fl-CUR-NPs predominantly localized in the cytoplasm, which may reflect endocytic uptake without nuclear entry. In contrast, MCF-7 cells (Figure 7) exhibited more rapid uptake, with NPs observed in close proximity to the nucleus (perinuclear region). After 1 h, the MDA-MB-231 cells showed gradual rounding and cytoplasmic volume reduction, features that could be associated with cytoskeletal stress and membrane remodeling during uptake. MCF-7 cells begin to exhibit nuclear compression and deformation with partial extrusion of intracellular components, implying stronger intracellular stress responses and possible lysosomal involvement [60,61]. At 3 h, the MDA-MB-231 cells displayed partial rupture and organelle extrusion, consistent with lysosomal membrane permeabilization and early apoptotic events [62,63]. In comparison, MCF-7 cells demonstrated widespread apoptotic body formation, a hallmark of apoptosis, together with visible membrane blebbing and cell shrinkage, indicating that apoptotic signaling was more rapidly activated in MCF-7 [63,64]. By 6 h, both cell types exhibited significant cytoplasmic compression and Fl-CUR-NP release, indicating increased membrane permeability. Notably, apparent organelle-associated changes and nanoparticle efflux were more pronounced in MCF-7 cells, which could reflect greater perturbation of intracellular trafficking or organelle stress, though the specific pathways involved remain to be determined [65]. At 24 h, the MDA-MB-231 cells appeared mostly rounded with fragmented nuclei and re-accumulated cytoplasmic NPs, indicative of late apoptosis. MCF-7, however, showed dense accumulation of apoptotic bodies around the nucleus, reflecting advanced apoptotic progression and extensive nuclear fragmentation.

Collectively, these morphological and subcellular observations correlated strongly with the results of cytotoxicity analysis (Figure 5) where MCF-7 exhibited a lower IC_50_ value than MDA-MB-231, confirming its higher susceptibility to Fl-CUR-NPs. This enhanced sensitivity could be attributed to faster NP uptake, perinuclear localization, and accelerated apoptotic activation.

It should be emphasized that the current evidence is primarily morphological and descriptive. Definitive mechanistic conclusions (e.g., specific involvement of lysosomal rupture, caspase-dependent apoptosis, mitochondrial dysfunction, or membrane permeabilization) cannot be drawn from these observations alone. To validate the proposed mechanisms, we plan to perform targeted biochemical and functional assays (e.g., Annexin-V/PI staining, caspase activation assays, JC-1 mitochondrial membrane potential measurements, and lysosomal integrity probes) in follow-up studies.

## 5. Conclusions

In summary, the developed CS/ALG/HA CUR-NPs effectively overcame the intrinsic limitations of CUR by improving its solubility, stability, and efficiency of cellular delivery. Compared with free CUR, the NPs exhibited optimal size distribution and stability, ensuring favorable biocompatibility and enhanced cytotoxicity. Moreover, cellular uptake studies revealed that the endocytic behaviors of MCF-7 and MDA-MB-231 cells were distinct, correlating with differences in apoptotic response. Collectively, these results indicated that NP-mediated subcellular targeting, particularly toward the nucleus, plays a pivotal role in enhancing anticancer efficacy. Therefore, this study not only provides a comprehensive understanding of cell line-dependent responses but also establishes a strategic framework for the rational design of CUR-based nanocarriers for precision cancer therapy.

## Figures and Tables

**Table 1:** Composition and description of CUR-NP formulations.

Abbreviation	CUR Amount Loaded (g)	CUR Concentration (*w*/*w*%)	Description
UL-NPs	0	0%	CUR unloaded-NPs (blank control)
CUR0.7-NPs	0.7	2.6%	NPs loaded with 0.7 g CUR (2.6% *w*/*w*)
CUR1.4-NPs	1.4	5.2%	NPs loaded with 1.4 g CUR (5.2% *w*/*w*)
CUR2.1-NPs	2.1	7.8%	NPs loaded with 2.1 g CUR (7.8% *w*/*w*)

## Data Availability

The original contributions presented in this study are included in the article/Supplementary Materials. Further inquiries can be directed to the corresponding author.

## Supplementary Materials

The following supporting information can be downloaded at: https://www.mdpi.com/article/10.3390/nano15221747/s1, Figure S1. Calibration curve of CUR constructed using UV–Vis spectrophotometry at 433 nm (0–1 μg/mL). The generated linear regression equation and R^2^ value confirm the high analytical accuracy of CUR quantification in NP samples. Figure S2. Particle size distribution profiles of CUR-NPs at different storage timepoints, (a) 0 days, (b) 21 days, (c) 35 days, (d) 110 days, and (e) 195 days. The results demonstrate that no significant broadening of particle size throughout the storage period, indicating the long-term stability of the CUR-NPs. Figure S3. Confocal fluorescence images showing cellular uptake of Fl-CUR-NPs after 3 months of storage. MDA-MB-231 cells were incubated for 6 h and MCF-7 cells for 3 h with the stored nanoparticles. Nuclei were stained with Hoechst 33342 (blue), and nanoparticle fluorescence is shown in green. Enlarged views highlight intracellular localization (red arrows). Comparable fluorescence intensity and distribution suggest that storage did not alter nanoparticle internalization efficiency. Scale bars = 20 μm. Figure S4. UV–Vis absorption spectra of CUR-NPs at different storage durations (0, 3, and 60 days). The characteristic absorption peaks of curcumin were preserved over time, indicating excellent drug retention and chemical stability during storage. Data are presented as mean ± SD (n = 5). Figure S5. The FTIR spectra of the raw materials: curcumin, chitosan, alginate, and hyaluronic acid (HA). Characteristic bands corresponding to each component are observed, confirming the presence of their respective functional groups prior to nanoparticle formulation. Figure S6. Representative gating strategy for flow cytometry analysis of CUR-NP uptake in breast cancer cells. (A–C) MDA-MB-231 and (D–F) MCF-7 cells. P1: initial cell population gated by size and granularity (FSC-A vs. SSC-A); P2: singlet discrimination based on SSC-H vs. SSC-A; P3: singlet confirmation based on FSC-H vs. FSC-A prior to fluorescence analysis. This gating workflow was consistently applied to all samples across different time points and experimental conditions.

## Abbreviations

ALG	Alginate
CS	Chitosan
CUR	Curcumin
DL	Drug loading
DMEM	Dulbecco’s modified Eagle’s medium
EE	Encapsulation efficiency
ER	Estrogen receptor
FBS	Fetal bovine serum
FTIR	Fourier-transform infrared
HA	Hyaluronic acid
NPs	Nanoparticles
PBS	Phosphate-buffered saline
SD	Standard deviation
SEM	Scanning electron microscopy
TEM	Transmission electron microscopy
TNBC	Triple-negative breast cancer
