# Supplementary material for: Curcumin-Loaded Polysaccharide Nanoparticles Enhance Aqueous Dispersibility and In Vitro Cytotoxicity in Breast Cancer Cell Lines
Source: Nanomaterials (Basel). 2025 Nov 20;15(22):1747. doi: 10.3390/nano15221747 (PMC12655040; doi:10.3390/nano15221747)
Supplement: Supplementary file 1 [file nanomaterials-15-01747-s001.zip › nanomaterials-3972043-supplementary.pdf]

Supplementary Materials

Curcumin-Loaded Polysaccharide Nanoparticles Enhance Aqueous Dispersibility and In Vitro Cytotoxicity in Breast Cancer Cell Lines

Yu-Chen Tsai [1], Hiroki Miyajima [1], Ming-Yang Chou [2,*], and Satoshi Fujita [1,*]

[1] Department of Frontier Fiber Technology and Sciences, University of Fukui, Fukui 910-8507, Japan

[2] ROHER Technology Co., Taichung 41141, Taiwan, Republic of China.

* Correspondence: rohertech007@gmail.com (M.Y.C.); fujitas@u-fukui.ac.jp (S.F.); Tel.: +81-0776-27-9969 (S.F.)

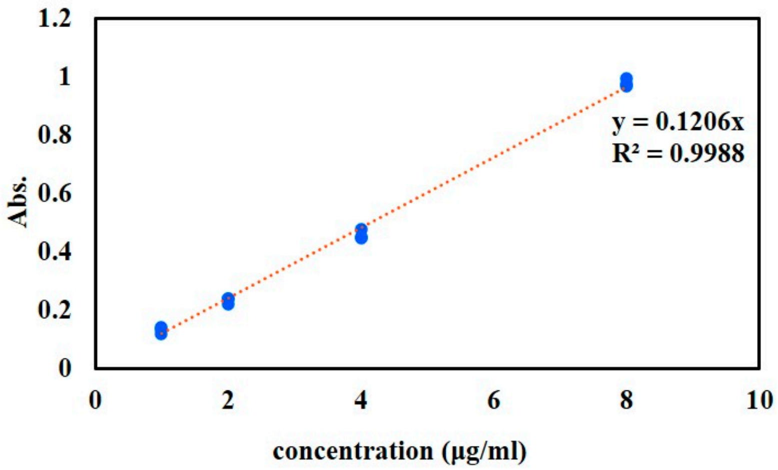

Figure S1. Calibration curve of CUR constructed using UV–Vis spectrophotometry at 433 nm (0–1 μg/mL). The generated linear regression equation and R^2 value confirm the high analytical accuracy of CUR quantification in NP samples.

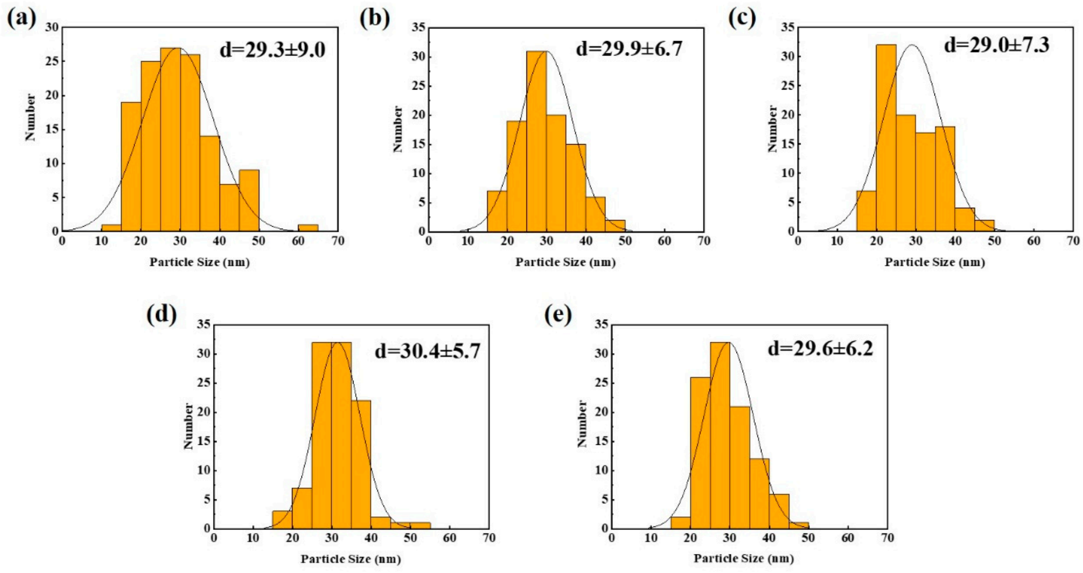

Figure S2. Particle size distribution profiles of CUR-NPs at different storage timepoints, (a) 0 days, (b) 21 days, (c) 35 days, (d) 110 days, and (e) 195 days. The results demonstrate that no significant broadening of particle size throughout the storage period, indicating the long-term stability of the CUR-NPs.

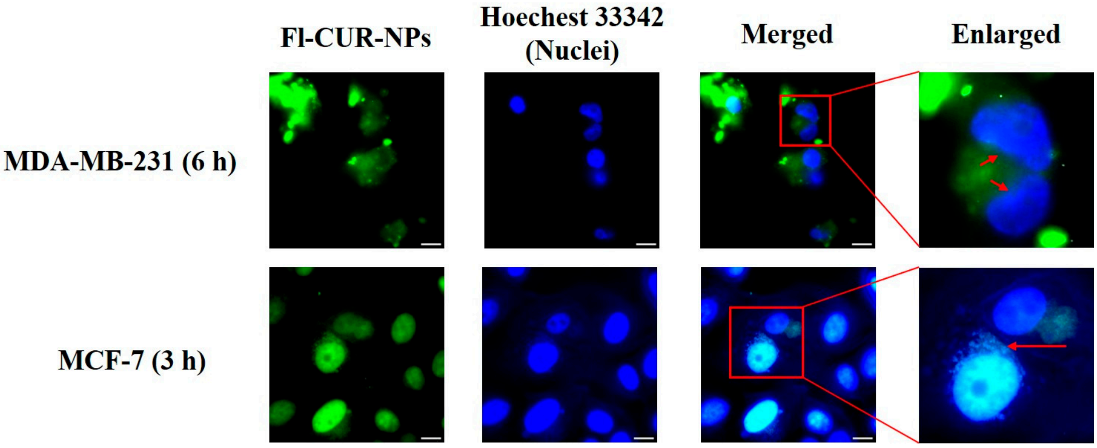

Figure S3. Confocal fluorescence images showing cellular uptake of Fl-CUR-NPs after 3 months of storage. MDA-MB-231 cells were incubated for 6 h and MCF-7 cells for 3 h with the stored nanoparticles. Nuclei were stained with Hoechst 33342 (blue), and nanoparticle fluorescence is shown in green. Enlarged views highlight intracellular localization (red arrows). Comparable fluorescence intensity and distribution suggest that storage did not alter nanoparticle internalization efficiency. Scale bars = 20 μm.

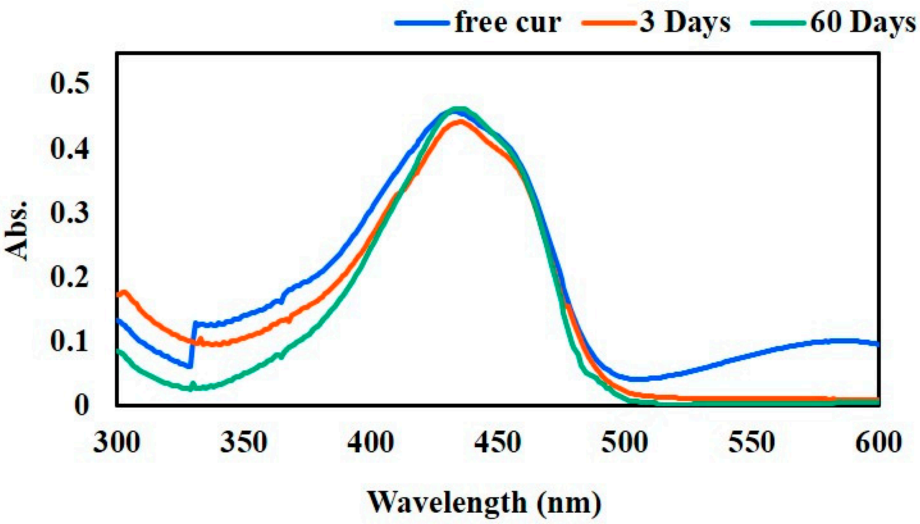

Figure S4. UV–Vis absorption spectra of CUR-NPs at different storage durations (0, 3, and 60 days). The characteristic absorption peaks of curcumin were preserved over time, indicating excellent drug retention and chemical stability during storage. Data are presented as mean ± SD (n = 5).

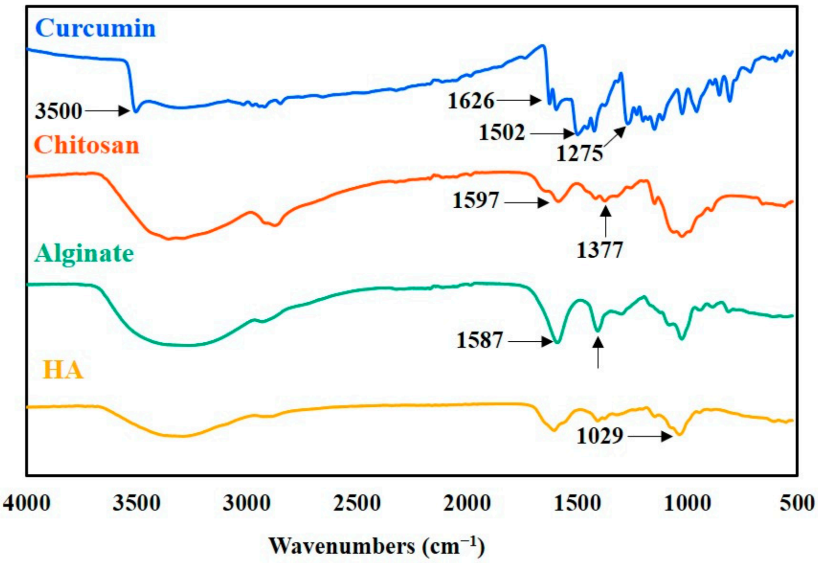

Figure S5. The FTIR spectra of the raw materials: curcumin, chitosan, alginate, and hyaluronic acid (HA). Characteristic bands corresponding to each component are observed, confirming the presence of their respective functional groups prior to nanoparticle formulation.

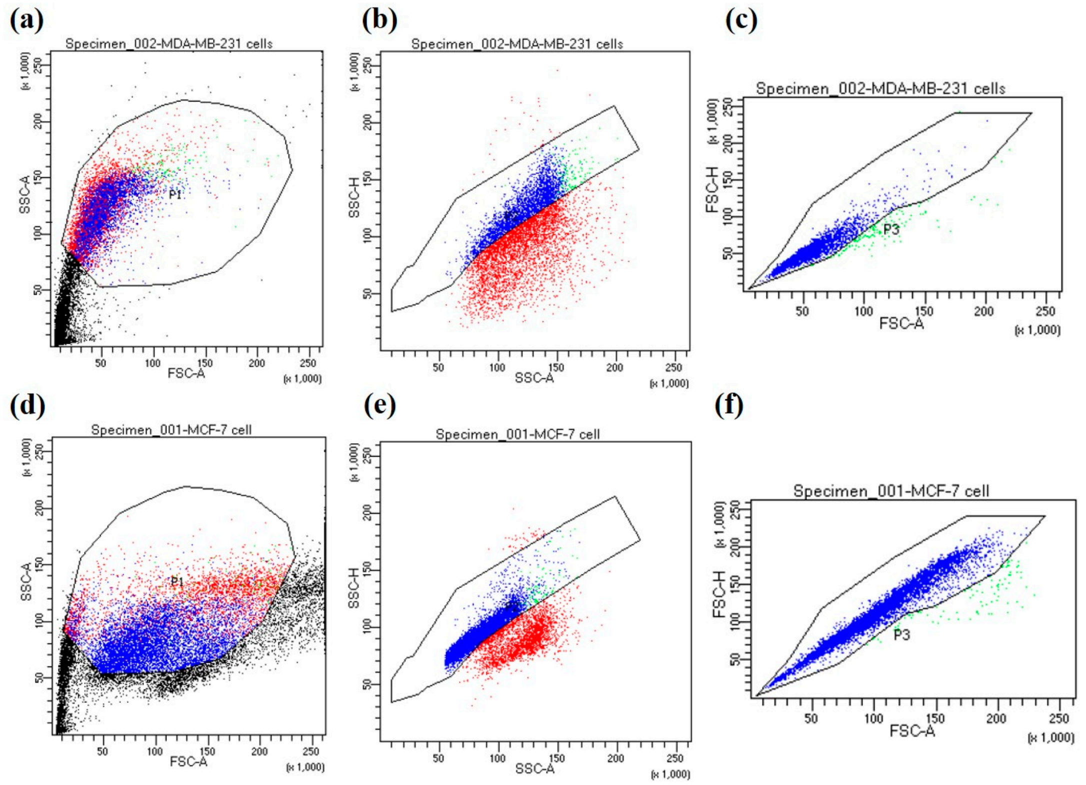

Figure S6. Representative gating strategy for flow cytometry analysis of CUR-NP uptake in breast cancer cells. (A–C) MDA-MB-231 and (D–F) MCF-7 cells. P1: initial cell population gated by size and granularity (FSC-A vs SSC-A); P2: singlet discrimination based on SSC-H vs SSC-A; P3: singlet confirmation based on FSC-H vs FSC-A prior to fluorescence analysis. This gating workflow was consistently applied to all samples across different time points and experimental conditions.
